# Combined wedge resection for lower lip debulking in Melkersson–Rosenthal syndrome

**DOI:** 10.1002/pdi3.74

**Published:** 2024-05-23

**Authors:** Hope Xu, Pranav N. Haravu, Russell R. Reid, Bruce Bauer

**Affiliations:** ^1^ Section of Plastic and Reconstructive Surgery Department of Surgery University of Chicago Medical Center Chicago Illinois USA

## 1 INTRODUCTION

Melkersson–Rosenthal syndrome (MRS) is a rare neuro‐mucocutaneous granulomatous disorder classically defined as a triad of recurrent orofacial edema, lingua plicata, and facial paralysis that present in the second and third decades of life.^1^ The full triad of symptoms has been quoted as occurring in only 8%–33% of patients, and given the heterogeneity in presentation, it often goes undiagnosed for years.^2^, ^3^ MRS tends to present similarly in two other conditions, granulomatous cheilitis (GC) and orofacial granulomatosis (OG), and as such is often discussed alongside them.^2^ The underlying pathology behind these disorders has yet to be elucidated, and suggestions range from local hypersensitivity reactions,^4^, ^5^ processes akin to other granulomatous diseases, such as sarcoidosis and Crohns disease,^6^, ^7^, ^8^, ^9^ and genetic predispositions evidenced by familial linkage and observed human leukocyte antigen overexpression.^10^, ^11^ Given the rare nature of the disease and a lack of clear etiology driving the disease process, treatments for MRS and associated GC/OG remain disjointed with no wide consensus and limited proven efficacy. A recent systematic review demonstrates that the literature is currently limited to individual case reports and small case series with no prospective studies. Furthermore, medical treatment described in the literature is heavily varied, including steroids, antibiotics, immunosuppressive therapy, and avoidance of suspected triggers.^2^

Though the presentation of MRS is heterogeneous, the lips are one of the most commonly affected areas with case series reporting 71%–96%^12^, ^13^ of patients exhibiting edema and swelling of the lips. Notably, medical management alone is often insufficient in correcting macrocheilia resulting in persistent esthetic and functional disturbances. This is likely partially driven by the irreversible soft tissue changes that occur as the disease develops as lymphoplasmacytic inflammatory and granulomatous infiltrates progress to fibrosis.^14^ Surgical management, specifically reduction cheiloplasty, has been shown to be an effective treatment for persistent medically refractory macrocheilia with satisfactory long‐term outcomes. Multiple surgical approaches have been described, including wedge resections,^15^ wedge resections with z‐plasties for fissures,^12^ fleur‐de‐lis resection with a transmodiolar labial suspension suture,^16^ transverse semilunar incisions (the Conway method) accompanied by tangential resection of the orbicularis,^17^, ^18^, ^19^, ^20^ and the Mouly method.^21^ Here, we present a case of a 20‐year‐old man who presented with enlargement and ptosis of the lower lip secondary to MRS and underwent surgical management via combined transverse and vertical wedge resection for his orofacial edema.

## 2 CASE REPORT

A 20‐year‐old man with no known past medical history presented for evaluation for a 3‐year history of recurrent lower lip swelling. When the swelling was first noted, it had been managed at an outside hospital with imaging and excision with pathology compatible with benign oral mucosa. However, the patient's lip swelling recurred shortly thereafter. He then attempted one round of sclerotherapy with diagnostic angiogram, which significantly exacerbated the swelling. He was subsequently referred to plastic surgery for further evaluation complaining of secondary ptosis causing irritation, dryness, and incompetence of the lip.

**FIGURE 1:**
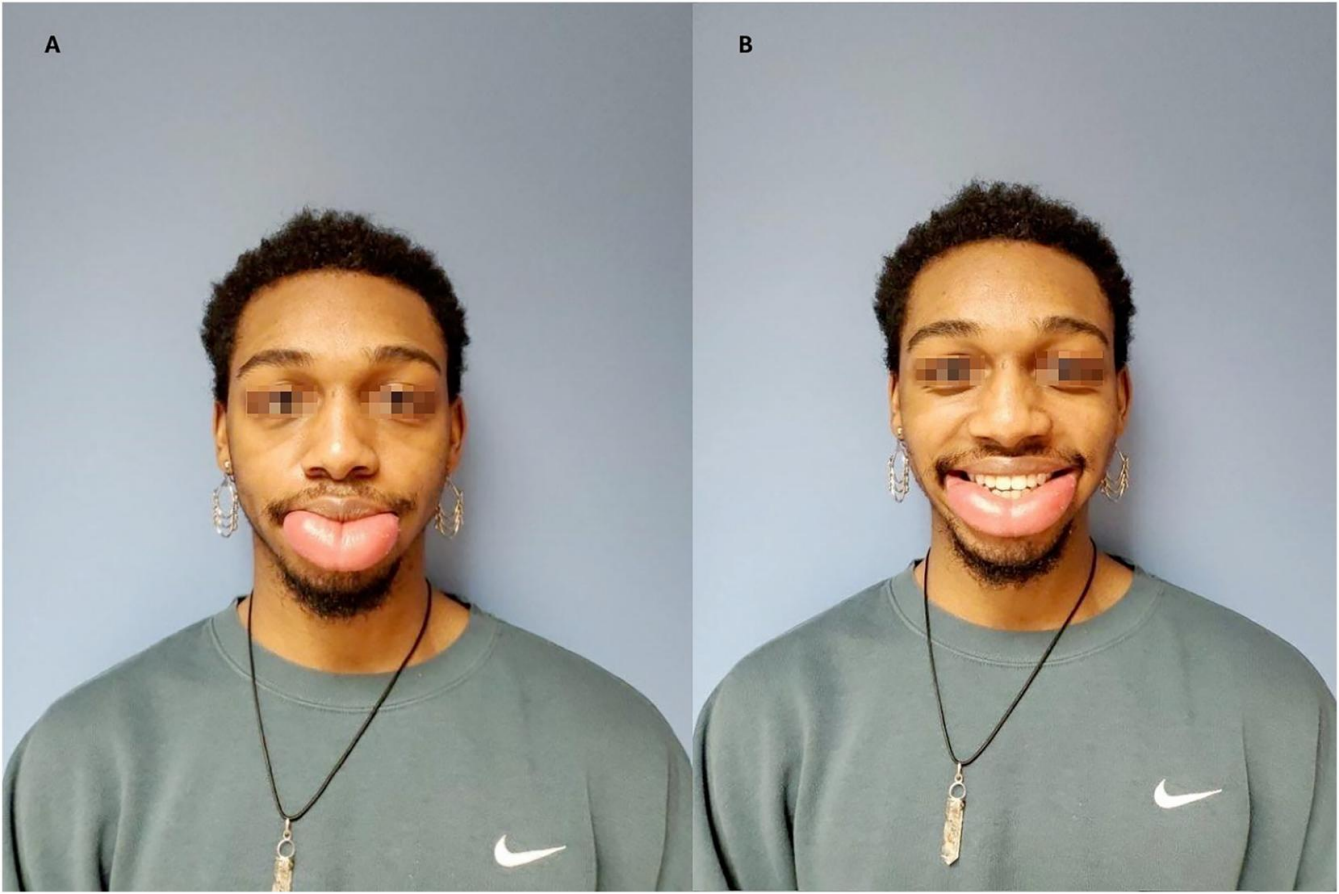
Preoperative photographs demonstrating diffuse lower lip edema with secondary ptosis (A) at rest and (B) with smile.

On physical examination, the patient demonstrated diffuse enlargement of the lower lip with denudation of the wet vermilion and mild Class III occlusion. There was severe lower lip edema and secondary ptosis (Figure 1A,B). Furthermore, there was evidence of tongue fissuring. Both facial and trigeminal nerve exams were full and intact bilaterally. After a history of angioedema or allergic symptoms was ruled out, the patient underwent imaging with MRI with contrast for further characterization. Imaging confirmed diffuse soft tissue swelling of the lower lip without clear radiologic evidence of etiology and a nonspecific mildly prominent right level 1 lymph node. Considering his refractory response to prior excision and conservative therapy, surgical debulking of the lower lip with possible preoperative embolization was planned.

**FIGURE 2:**
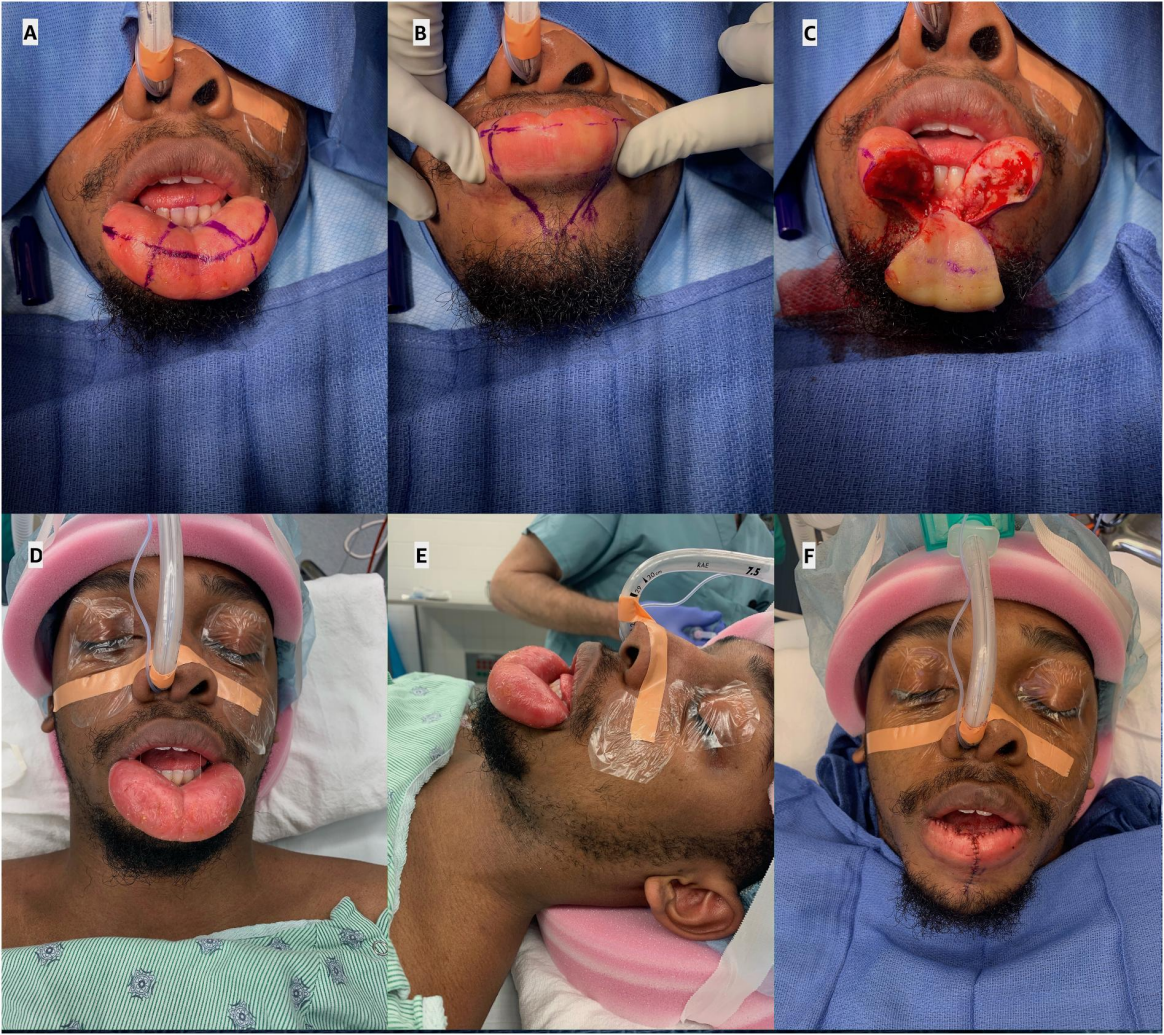
Intraoperative photos for (A) planned transverse semilunar and (B) vertical (central) wedge resections, (C) first stage full thickness vertical (central) wedge resection and side‐by‐side comparison of (D, E) preoperative, and (F) postoperative lower lip after resection and repair.

The patient was referred to neurointerventional radiology for neuroangiogram and was found to have large branches from the lingual artery bilaterally entering the lower lip with no evidence of shunting, arteriovenous malformation, or tumor blush. The patient proceeded to planned surgery 1 week later. Surgical debulking was performed via combined vertical and transverse wedge resections. The degree of central wedge resection was first marked out measuring 2.5–3.0 cm in width, with an accompanying transverse semilunar excision (Figure 2A,B). The vertical wedge resection was carried out through the full thickness of the lip (Figure 2C), and initial muscle and deep dermal sutures were placed to reapproximate the vermilion‐cutaneous junction. Next, the transverse wedge resection was defined and flaps were extended out from the midline toward each commissure. A broad triangle of infiltrative process was noted deep at the level of orbicularis oris and mucosa, which was included in the resection through the full vertical height of the lip. The excess tissue was advanced and resected, and closure was aligned such so that all four segments of the flap did not meet at a single point. A multilayered closure was completed to minimize tension on the mucosa and flaps (Figure 2D–F). Final pathology demonstrated extensive fibrosis and scattered non‐necrotizing granulomas positive for CD68 on immunohistochemistry consistent with MRS.

**FIGURE 3:**
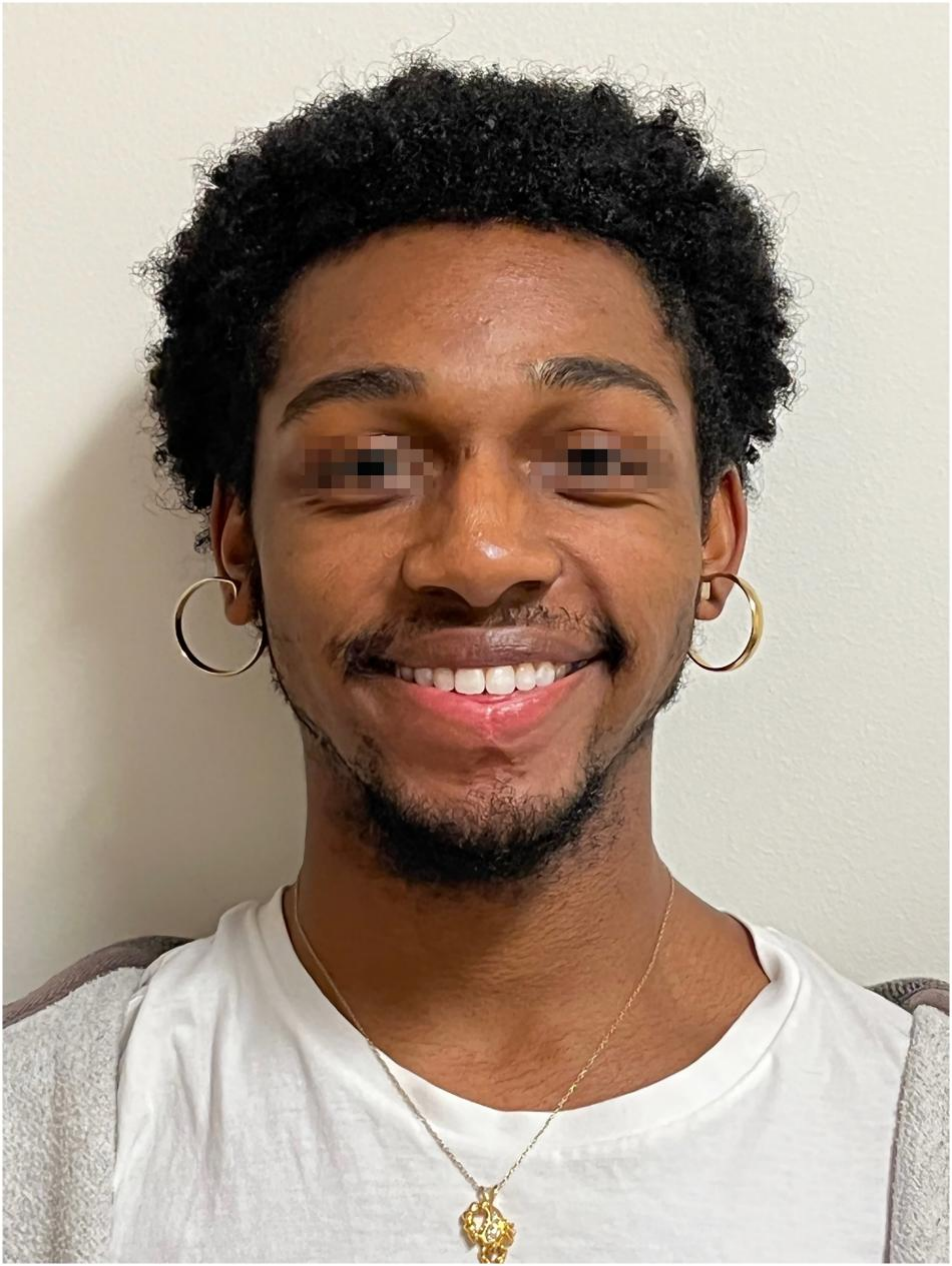
Postoperative photographs obtained at 12‐month follow‐up.

At the 1‐week and 3‐week follow‐up, there was a marked reduction in lower lip volume and wet lip show. Oral continence, labial sensation, and lip movement all improved rapidly with intact repair. At the 2‐month follow‐up, the patient noted early signs of expected scar contracture at the vermilion‐cutaneous junction, and at 6 months, he began receiving kenalog injections to assist with scar resolution. He completed three rounds of injections at his 1‐year follow‐up (Figure 3). At his 16‐month follow‐up, the patient opted to undergo a final z‐plasty revision at the labiomental fold under local anesthesia to optimize scar contour. Throughout his recovery, the patient's lip remained stable in size with no healing issues or signs of recurrence.

## 3 DISCUSSION

Melkersson–Rosenthal syndrome most commonly presents mono‐symptomatically and rarely presents with the full classic triad it is known for.^22^ This lends complexity to the process of diagnosing and treating individuals who suffer isolated symptoms as they are often referred to multiple specialists and undergo a spectrum of treatment modalities with otolaryngologists, dermatologists, plastic surgeons, and ophthalmologists for seemingly unrelated issues. Though our patient's presentation was not complicated by the addition of facial paralysis, a final diagnosis and opportunity for genetic counseling was still delayed by multiple consultations, rounds of imaging, and both surgical and nonsurgical interventions.

Though a clear understanding of the etiology behind MRS remains elusive, surgical management has been demonstrated to play a crucial role in persistent and refractory orofacial swelling. Lip swelling that has progressed to a degree involving infiltrative fibrosis and granulomatosis cannot be structurally undone with noninvasive methods as seen in the case of our patient here. The goal of surgical management of the MRS lip should be to debulk to improve lip function, restore lip symmetry, and resect affected mucosa and/or muscle to prevent recurrence. The reduction cheiloplasty discussed in this case report can be best described as a modified Conway procedure, featuring a transverse mucosal incision dorsal to the vermilion border combined with a central wedge excision, addressing both bulk and infiltrated mucosa.^17^ A similar approach has been described in a case report by Azimi et al. for a patient suffering from upper lip swelling with confirmed MRS^19^ featuring an extended resection of the orbicularis oris and mucosa for redundancy. In their report, the patient enjoyed similar improvements in volume, cosmesis, oral continence, and sensation at the 5‐month follow‐up, solidifying the efficacy of the described approach for both upper and lower lip swelling. Furthermore, the extended and frequent degree of follow‐up documented in our experience underscores the longevity of our patient's outcome and his associated satisfaction in the absence of major revisions.

## 4 CONCLUSION

Melkersson–Rosenthal syndrome is a rare and underdiagnosed disease process that remains difficult to identify and treat. The case presented here underscores the value of timely diagnosis and surgical excision in resolving persistent macrocheilia refractory to noninvasive treatments due to chronic granulomatosis and fibrosis. Appropriate planning of the reduction cheiloplasty should address bulk, symmetry, and degree of infiltration to produce a lasting result balancing cosmesis and function.

## AUTHOR CONTRIBUTIONS

**Hope Xu**: Organization and drafting of manuscript and revisions, as well as figure assembly. **Pranav Haravu**: Drafting of manuscript. **Russell R. Reid**: Co‐surgeon, manuscript review. **Bruce Bauer**: Senior surgeon, manuscript review.

## CONFLICT OF INTEREST STATEMENT

Russell R. Reid is a member of Pediatric Discovery Editorial Board. To minimize bias, he was excluded from all the editorial decision‐making related to the acceptance for publication. The other authors declare no conflicts of interest.

## ETHICS STATEMENT

The patient presented in this study completed informed consent, photographic consent, and consent for publication. All data reported in this manuscript is in compliance with approved IRB protocol and standard ethical principles. Per University of Chicago Medicine Institutional Review Board Guidelines, this case report study was exempt from committee review and therefore approved for publication.

## Data Availability

Data sharing is not applicable to this article as no new data were created or analyzed in this study.
